# Editorial: Role of ncRNAs in immunogenic cell death of cancer

**DOI:** 10.3389/fimmu.2026.1788538

**Published:** 2026-02-11

**Authors:** Shaochu Zhuo, Tong Zhu, Nan Zhao, Fu Peng, Xudong Zhu, Zhiyu Wang, Neng Wang

**Affiliations:** 1 State Key Laboratory of Traditional Chinese Medicine Syndrome/Research Centre of Basic Integrative Medicine, School of Basic Medical Sciences, Guangzhou University of Chinese Medicine, Guangzhou, Guangdong, China; 2 Guangdong Provincial Key Laboratory of Clinical Research on Traditional Chinese Medicine Syndrome, Guangdong Provincial Academy of Chinese Medical Sciences, The Second Affiliated Hospital of Guangzhou University of Chinese Medicine, Guangzhou, Guangdong, China; 3 Department of Breast Surgery, Panjin Central Hospital, Panjin, China; 4 Department of Hepatopancreatobiliary Surgery, Cancer Hospital of Dalian University of Technology, Cancer Hospital of China Medical University, Liaoning Cancer Hospital and Institute, Shenyang, Liaoning, China; 5 West China School of Pharmacy, Sichuan University, Chengdu, China

**Keywords:** cancer immunotherapy, immunogenic cell death (ICD), non-coding RNA (ncRNA), RNA crosstalk, tumor microenvironment

## Immunogenic cell death in cancer therapy

Cancer remains a complex, heterogeneous disease, constituting a major global health burden. While advances in surgery, radiotherapy, chemotherapy, and targeted therapies have reshaped clinical practice, enduring challenges such as recurrence, metastasis, and drug resistance continue to impede curative outcomes. In recent years, immunotherapy — largely driven by immune checkpoint inhibitors (ICIs) — has introduced a paradigm shift in cancer treatment. Nevertheless, its efficacy is still limited to a subset of patients, making the improvement of response rates and long-term efficacy a central focus of contemporary research. Within this context, ICD has emerged as a promising therapeutic frontier (1). By triggering the release of damage−associated molecular patterns (DAMPs) through distinct molecular mechanisms, ICD converts dying tumor cells into potent inducers of adaptive immune activation (2).

Parallel advances in non-coding RNA (ncRNA) research have unveiled a new dimension for deciphering the complexity of biological regulation. Once considered as “genomic dark matter”, molecules such as microRNAs (miRNAs), long non-coding RNAs (lncRNAs), and circular RNAs (circRNAs) are now recognized as key regulators within intricate transcriptional and post-transcriptional networks (3), with a profound influence on processes such as cell fate determination and immune microenvironment modulation (4). A single ncRNA can often modulate hundreds of downstream targets, establishing these molecules as potential “molecular hubs” for intervening in ICD-associated pathways and reshaping the tumor immune microenvironment. However, research on ICD-associated ncRNAs currently faces several critical gaps, including a lack of systematic screening, unclear regulatory hierarchies and interaction networks among different ncRNA types, and unrealized potential for clinical translation as biomarkers or therapeutic targets.

## Advances in ICD-associated ncRNA regulation

**Figure 1:**
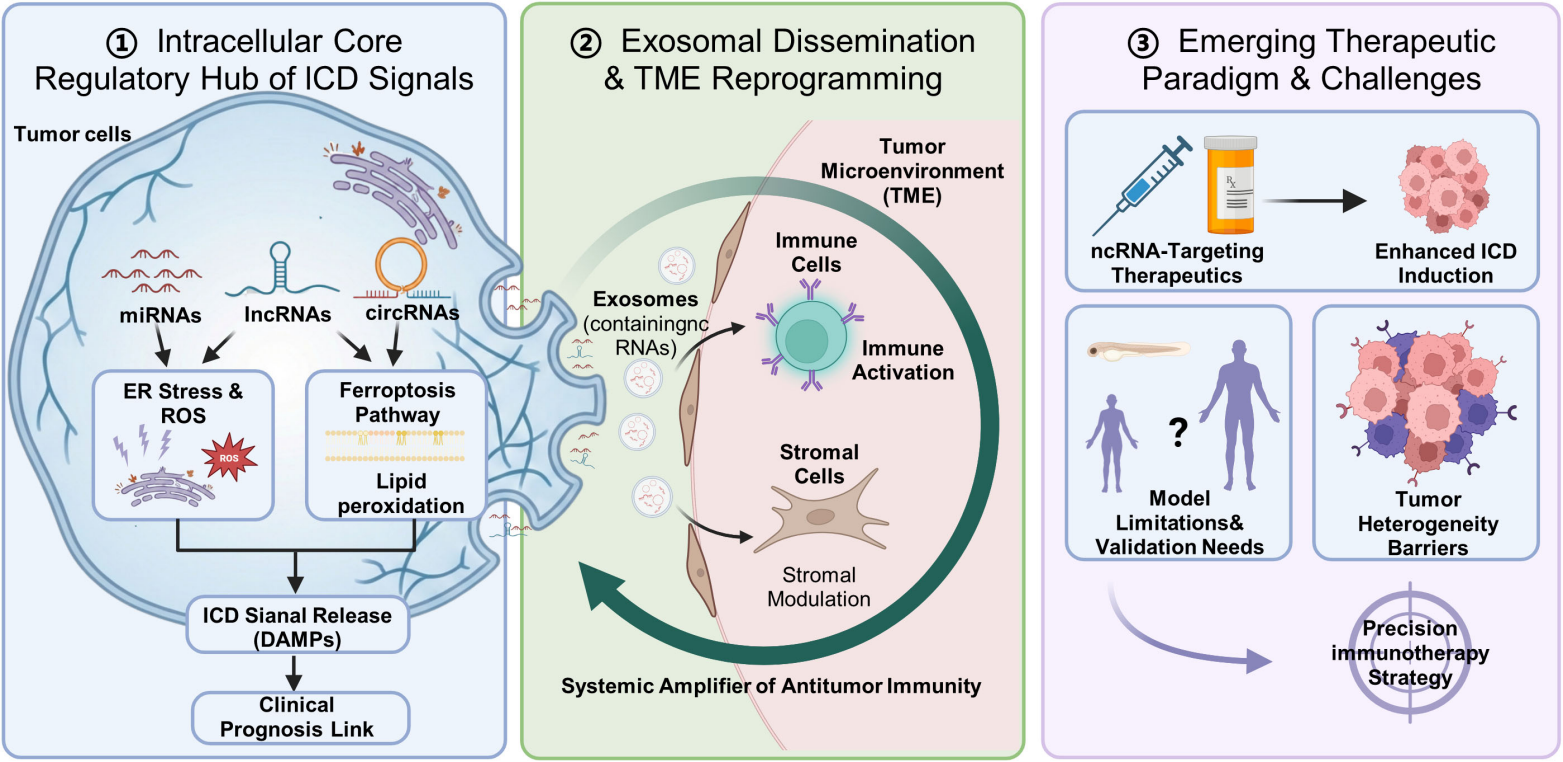
The pivotal role of non-coding RNA (ncRNA) in the Immunogenic cell death (ICD)–tumor immunity axis. ①ncRNAs serve as central regulators of ER stress, ROS, ferroptosis, and related processes to initiate ICD signaling. ②Exosome-encapsulated ncRNAs reprogram the tumor microenvironment (TME) through immune cell activation. ③Emerging ncRNA-targeted strategies and their challenges (e.g., model validation, tumor heterogeneity) are outlined.

This Research Topic focuses on the “ncRNA — ICD — tumor immunity” regulatory axis, addressing a central scientific question: How do ICD-related ncRNAs precisely regulate cancer progression? It delineates how miRNAs rapidly orchestrate ICD, how lncRNAs epigenetically regulate its potential, and how circRNAs mediate ICD transmission by acting as miRNA sponges (5). The collected studies analyze the pivotal role of ncRNAs within the ICD — tumor immunity axis from three distinct perspectives (**Figure 1**). In the sections that follow, we will elaborate on these findings based on the six articles included in this column.

Firstly, ncRNAs constitute the core regulatory hub of the ICD signaling network, and the articles in this column systematically reveal how they execute this pivotal function to precisely regulate cancer progression through mechanistic insight, functional expansion, and clinical validation. Fang et al. elucidated that diverse ncRNAs acted as precision tuners of ICD by directly modulating core pathways such as endoplasmic reticulum stress and ROS production, thereby determining the strength and specificity of immunogenic signals and providing a mechanistic framework for understanding miRNA-mediated modulation and circRNA sponging effects. Furthermore, Ju et al. revealed that lncRNAs could epigenetically regulate ferroptosis — a cell death modality sharing overlapping pathways with ICD — thereby expanding the scope and depth of ncRNA-mediated immunogenic stress responses and underscoring their functional diversity and synergy. The clinical significance of these foundational mechanisms is concretely demonstrated by Yan et al. Their large-scale meta-analysis established a robust association between ICD-driven immune infiltration and cancer patient prognosis, thereby completing the critical translational link from ncRNA-regulated molecular events to clinically observable anti-tumor immunity.

Secondly, exosomal ncRNAs serve as key mediators in disseminating the immune effects of ICD and the reprogramming of the tumor microenvironment. This column examined how ncRNAs extend beyond cell-autonomous regulation by leveraging extracellular vesicles for intercellular communication, thereby systemically disseminating ICD−induced immune responses. Duan et al. demonstrated that tumor−derived exosomal ncRNAs (e.g., miRNAs, circRNAs) functioned as active signaling mediators that reprogrammed the tumor microenvironment and remotely modulated immune or stromal cell activity. These findings provided essential spatiotemporal and mechanistic context for processes such as circRNA−mediated miRNA sponging in ICD transmission, illustrating how ncRNAs serve as systemic amplifiers that convert local ICD events into broad antitumor immunity.

Thirdly, targeting ICD-associated ncRNAs, despite challenges such as model limitations and tumor heterogeneity, has emerged as a promising new paradigm for cancer immunotherapy. Addressing this therapeutic direction, the column provided in-depth discussions at the conceptual, methodological, and critical levels. Sun et al. explicitly proposed and substantiated this strategy, which builds precisely on the earlier elucidated mechanisms of ncRNA-mediated fine-tuning, aiming to enhance ICD and thus improve immunotherapy outcomes by modulating specific miRNA, lncRNA, or circRNA networks. To advance the translation of this paradigm, Hu et al. highlighted the unique strengths of the zebrafish model for real-time, ex *vivo* functional studies of ncRNAs and drug screening, offering an innovative tool to address tumor heterogeneity and dynamically evaluate ncRNA-targeting interventions. Collectively, however, these articles also pointed to persistent challenges, including the need for more precise disease models to validate targets and the necessity of overcoming tumor heterogeneity to enable ncRNA-stratified precision intervention. These considerations outline critical pathways for translating fundamental insights from the “ncRNA — ICD — immunity” axis into clinical practice.

## Conclusions and perspectives

In summary, the studies presented in this column establish ncRNAs as a strategic hub linking ICD with anti-tumor immunity. Through orchestrating intracellular networks and enabling intercellular signaling, ncRNAs play a decisive role in launching and amplifying the anti-tumor immune response (6). Future research may focus on three key directions: clarifying the spatiotemporal expression and functional dynamics of ncRNAs during ICD; constructing experimental models that authentically mimic the tumor microenvironment to replicate ncRNA regulatory networks; and advancing targeted delivery and modulation strategies to overcome tumor heterogeneity and achieve individualized immunoenhancement. Addressing these challenges will propel the field from molecular identification toward network-level programming, ultimately harnessing the regulation of “RNA crosstalk” to open new frontiers in cancer immunotherapy.
